# 4-Acetylantroquinonol B inhibits lipopolysaccharide-induced cytokine release and alleviates sepsis through of MAPK and NFκB suppression

**DOI:** 10.1186/s12906-018-2172-2

**Published:** 2018-03-23

**Authors:** Chien-Hsin Chang, Chun-Chieh Hsu, An-Sheng Lee, Shih-Wei Wang, Kung-Tin Lin, Wei-Luen Chang, Hui-Chin Peng, Wen-Chiung Huang, Ching-Hu Chung

**Affiliations:** 1 Institute of Pharmacology, College of Medicine, National Taiwan University, Taipei, Taiwan; 2 Medical and Pharmaceutical Industry Technology and Development Center, New Taipei City, Taiwan; 3 Department of Medicine, Mackay Medical College, No. 46, Sec. 3, Jhong-Jheng Rd., Sanzhi Dist, New Taipei City, Taiwan; 4 Department of Pharmacology and Toxicology, Tzu Chi University, Hualien, Taiwan

**Keywords:** Antrodia cinnamomea, 4AAQB, Anti-inflammation, MAPK, NFκB

## Abstract

**Background:**

*Antrodia cinnamomea* is an indigenous medicinal mushroom in Taiwan, commonly used for the treatment of cancers and inflammatory disorders. 4-acetylantroquinonol B (4AAQB) is one of the active component isolated from the mycelium of A. *cinnamomea*. However, whether 4AAQB exhibits anti-inflammatory effect is not clear.

**Methods:**

The anti-inflammatory activity of 4AAQB was examined by ELISA to measure the pro-inflammatory cytokines production in lipopolysaccharide (LPS)-simulated RAW264.7 cells, peritoneal macrophages and in mice. The effect of 4AAQB for MAPK kinase molecules phosphorylation in LPS-stimulated RAW264.7 macrophage including ERK, JNK and p38 were evaluated. The in vivo efficacy of 4AAQB was also demonstrated.

**Results:**

In the present study, we found that 4AAQB exhibits anti-inflammatory effects inhibit tumor necrosis factor-α (TNF-α)/interleukin-6 (IL-6) releasing and LPS-stimulated phagocytes migration without affect cell growth. In addition, the MAPK kinase molecules phosphorylation in LPS-stimulated RAW264.7 macrophage including ERK, JNK and p38 was inhibited by 4AAQB. The phosphorylation of NFκB subunit p65 and IkBα were also decreased after 4AAQB treatment. Furthermore, 4AAQB attenuates the cytokine production in LPS-induced and CLP-induced septic mice.

**Conclusion:**

These results showed that 4AAQB exhibited anti-inflammatory property both in vitro and in vivo, suggesting that 4AAQB may be a therapeutic candidate which used in inflammatory disorders treatment.

**Electronic supplementary material:**

The online version of this article (10.1186/s12906-018-2172-2) contains supplementary material, which is available to authorized users.

## Background

Inflammation is an adaptive response which is triggered by harmful stimuli such as tissue injury and infection [1, 2]. In response to invading pathogens, macrophages activate and release proinflammatory cytokines (TNF-α, IL-6, IL-1β) and proinflammatory mediators like cyclooxygenase (COX)-2, and nitric oxide (NO) [3]. Overproduction of these pro-inflammatory cytokines and mediators causes sustained inflammation which can lead to numerous diseases like atherosclerosis, cancer, and cardiovascular disease.

Sepsis is a systemic inflammatory response and finally resulting in multiple organ dysfunction, refractory hypotension, and high mortality [4]. Lipopolysaccharide (LPS) was an endotoxin derived from gram-negative bacteria which interacts with monocytes/macrophages and lead to the production of pro-inflammatory cytokines and mediators [5]. Toll-like receptor 4 (TLR-4) recognizes LPS and leads to the activation of intracellular signaling proteins, including mitogen-activated protein kinase (MAPK) and Nuclear Factor-κB (NFκB) [6].

*Antrodia cinnamomea* (A. *cinnamomea*) is a traditional medicine and a unique fungus originating from Taiwan. This Chinese medicinal herb was widely used for abdominal pain, inflammatory disorders, liver diseases, and cancer treatment [7, 8]. Previous studies have shown that A. *cinnamomea* exhibits various pharmacological effects including anti-cancer, anti-inflammatory and antioxidative and hepatoprotective activities [7, 9–11]. 4-acetylantroquinonol B (4AAQB), isolated from the mycelium of A. *cinnamomea*, was previously demonstrated to inhibit the growth of hepatocellular carcinoma cells [12]. Recent studies showed that 4AAQB exhibited inhibitory effects on hepatoma cell growth via cell cycle and PI3K/Akt/mTOR pathways inhibition, and further displayed prominent anti-tumor growth and its metastatic in vivo [13, 14]. While 4AAQB has been shown previously to inhibit nitric oxide production induced by LPS in murine macrophages [15], the molecular mechanisms underlying the effect produced by 4AAQB have not been characterized so far. Moreover, there are very limited studies investigate the anti-inflammation efficacy in in vivo models about 4AAQB and other related antroquinonol derived. In this study, we found that 4AAQB may modulate the MAP kinases and NFκB signaling pathways and decrease inflammatory cytokines to possess its anti-inflammatory effects. Furthermore, 4AAQB displayed prominent anti-inflammation effects in LPS and CLP-induced sepsis mice models.

## Methods

### Plant material

*Antrodia cinnamomea* (BCRC35716) was obtained from the Bioresources Collection and Research Center (BCRC) (Hsinchu, Taiwan). A voucher specimen (MMC-002) was deposited in the Department of Medicine, Mackay Medical College (New Taipei City, Taiwan). 4-Acetylantroquinonol B was isolated from the mycelium of A. *cinnamomea* as described previously [12]. After the end of the fermentation process, the mycelial ethanol extract was re-dissolved in water and extracted with ethyl acetate. The water layer was then extracted with water saturated n-butanol and was further purified by silica gel chromatography and high-performance liquid chromatography. The structure of the 4AAQB was identified by nuclear magnetic resonance (NMR; AMX-400, Bruker, Billerica, MA, USA) and the detail information about identifying 4AAQB was shown in Additional file 1.

### Chemical materials

The enzyme-linked immunosorbent assays (ELISA) kit for TNF-α and IL-6 were from eBioscience (San Diego, CA, USA). DMEM, FBS, and all culture reagents were purchased from Gibco BRL (Life technologies, USA). Antibodies against p38, phosphorylated p38, JNK, phosphorylated JNK, ERK, phosphorylated ERK, α-tubulin, iNOS, phosphorylated NFκB p65 were purchased from Santa Cruz (Biotechnology, Inc., USA). β-actin antibody was from Novus Biologicals (Littleton, CO, USA). Antibodies against phosphorylated IκBα, phosphorylated STAT1 were purchased from Cell Signaling Technologies (Boston, MA, USA). Lipopolysaccharide (LPS, from *E. coli*, 0127: B8), gelatin, dexamethasone (Dex) and other chemicals were purchased from Sigma Chemicals, Co. (St Louis, Mo, USA).

### Cell cultures

The RAW264.7 macrophage cell line was obtained from American Type Culture Collection. The cells were cultured at 37 °C in HEPES-buffered Dulbecco’s modified Eagle’s medium (DMEM), containing 10% fetal bovine serum (Gibco) supplemented with NaHCO3, glutamax I (Gibco), 100 IU/mL penicillin G (sodium salt),100 μg/mL streptomycin, and 0.25 μg/mL amphotericin B (antibiotic−antimitotic solution, Gibco). Murine peritoneal macrophage cells were elicited by intraperitoneal injection of 1 ml of 4% (*w*/*v*) thioglychollate into the peritoneal cavity of 4 weeks old BALB/c male mice [16]. The animals were maintained on a 12-h light/dark cycle under controlled temperature (20 ± 1 °C) and humidity (55 + 5%) and were given continuous access to food and water. After 5 days, peritoneal exudates cells were obtained by lavage with 10 ml ice-cold DMEM. The harvested cells were treated with ACK lysis buffer (0.15 M NH4Cl, 10 mM KHCO3, 0.1 mM EDTA) for 1 min to lyse the red blood cells. After removing the ACK lysis buffer, the cells were washed twice and resuspended in HEPES-buffered DMEM and were seeded in sterile disposable culture plates.

### Cell viability assay

Cell viability was determined by 3-(4,5-dimethyl-thiazol-2-yl)-2,5-diphenyl tetrazolium bromide (MTT; Sigma, St Louis, MO, USA) reduction assay. In brief, RAW264.7 cells and murine peritoneal macrophage cells were preincubated overnight in 48-well plates at a density of 2 × 10^5^ cells/well (murine peritoneal macrophage cells) of 5 × 10^4^ cells/well (RAW264.7 cells) and were then treated with various concentrations of 4AAQB coexisted with LPS (100 ng/mL). After LPS stimulation for 24 h, the culture supernatants were replaced with MTT (0.5 mg/ml) at 37 °C with 5% CO2 for 30 min. The resulting dark blue crystals were then dissolved in DMSO (200 ul/well) after the MTT was removed. Absorbance values were read at 550 nm with an automated Spectra MAX 340 (Molecular Devices, Sunnyvale, CA, USA). All determinations were obtained by replication in at least three independent experiments.

### Cytokine assays

RAW264.7 cells and murine peritoneal macrophage cells were plated onto 48-well plates at a density of 2 × 10^5^ cells/well, and then treated with various concentrations of 4AAQB or positive control (Dexamethasone, Dex) coexisted with LPS (100 ng/ml) for 24 h. The cell supernatants were collected by centrifugation. Cytokines were measured by ELISA kit according to the manufacture’s instruction.

### Migration assay

Migration assay of murine peritoneal macrophage cells was measured by Coaster Transwells (polycarbonate filter, 5 μm pore size), which were coated with 0.2% gelatin. Murine peritoneal macrophage cells (1 × 10^5^ cells/well) were incubated with vehicle or different concentrations of 4AAQB at 37 °C for 30 min and then treated with LPS (100 ng/ml), and cells were seeded into the upper chamber. After incubation at 37 °C for 20 h, the macrophages that had transmigrated and bound to the membrane were fixed with 4% paraformaldehyde and stained with 0.5% toluidine. Migration was quantified by counting the number of stained cells with light microscope (Nikon, Japan) in 3 random fields (200×) and then photographed. All determinations were obtained by replication in at least three independent experiments.

### Nitric oxide assay

The nitrite accumulated in the culture medium was measured as an indicator of nitric oxide (NO) production based on the Griess reaction [17]. In brief, RAW264.7 cells were treated with or without LPS (100 ng/ml) in the presence of various concentrations of 4AAQB. After 24 h, culture supernatants were mixed with Griess reagent [equal volumes of 1% (*w*/*v*) sulfanilamide in 5% (*v*/v) phosphoric acid and 0.1% (w/v) naphtylethylenediamine–HCl], and incubated at room temperature for 10 min. Absorbance values were read at 550 nm and NO concentration was calculated with reference to a standard curve of sodium nitrite.

### Western blot analysis

RAW 264.7 cells (2 × 10^5^ cells/ml) and murine peritoneal macrophage cells were plated onto 6-well plates and pretreated with 4AAQB for 30 min and then stimulated with the presence or absence of 100 ng/ml LPS for 6 h (for iNOS), 30 min (for MAPKs and IκBα). Aliquots of total cell lysates or cell membrane fractions were analyzed by Western blotting. For quantification of the Western analysis, the density of each band was quantified by ImageJ software.

### Animals

Male ICR mice was obtained from BioLASCO Taiwan. Male ICR mice weighing 25–30 g were used in the animal model. All the experimental protocols regarding animal studying have been approved by the Laboratory Animal Use Committee of MacKay Medicine College.

### LPS-induced endotoxemia

Mice were anesthetized with inhalation anesthetic drug (Isoflurane) and injected intraperitoneally (i.p.) with DMSO (as control, 20 μl) or 4AAQB (0.3, 1 and 3 mg/kg) (10 mice in each group). Mice were injected intraperitoneally (i.p.) with LPS (20 mg/kg) 30 mins later. Mice were sacrificed 24 h after LPS and drug treatment. The health of mice was monitored every 6 h and record the mice number of survival. After experiment, all animals were euthanasia with CO2.

### Cecal Ligation Puncture (CLP)-induced sepsis

Mice were anesthetized with inhalation anesthetic drug (Isoflurane,) and then injected intraperitoneally (i.p.) with DMSO (as control, 20 μl) or 4AAQB (0.3 and 3 mg/kg) (13 mice in control group and 8 mice each in 4AAQB (0.3 and 3 mg/kg) group). Next, a 2-cm midline incision was performed to allow exposure of the cecum with adjoining intestine. The cecum was tightly ligated with a 3.0-silk suture at 1 cm from the cecal tip and punctured once with a 23-gauge needle. The cecum was then gently squeezed to extrude a few feces from the perforation sites and returned to the peritoneal cavity. The laparotomy site was the stitched with 4.0 silk. In control mice, the cecum was exposed but not ligated or punctured and then returned to the abdominal cavity. The health of mice was monitored every day and record the mice number of survival. After experiment, all animals were euthanasia with CO2.

### Mice whole blood and serum collection

After injecting intraperitoneally (i.p.) with LPS or CLP operation for 24 h, mice were anesthetized by i.p. injection of sodium pentobarbital (50 mg/kg) and whole blood was drawn by cardiac puncture or from the eye holes and collected in citric acid-citrate dextrose (ACD; 9:1 blood *v*/v) at indicated time. The sera were obtained by centrifugation at 1000×g for 10 min. After these studies finished, we used carbon dioxide (CO_2_) overdose to euthanasia for all animals.

### Measurement of cytokine levels

The concentration of cytokines, TNF-α and IL-6, in the sera was determined by ELISA kit according to the manufacture’s instruction.

### Histological examination

Lung, liver, kidney segments were cut off and fixed in 10% (*v*/v) phosphate-buffered formalin for 48–72 h and then embedded in paraffin. Next, the samples were sectioned (5 mm) using a microtome, stained with H&E, and examined with light microscopy at 400× magnifications.

### Statistical analysis

All values are presented as mean ± S.E.M. Differences between groups were assessed by one-way ANOVA and Newman-Keuls multiple comparison tests where appropriate. *P* values less than 0.05 (*p* < 0.05) were considered as significant difference.

## Results

### Effects of 4AAQB on cell viability in murine macrophage cell line and peritoneal macrophage

**Fig. 1:**
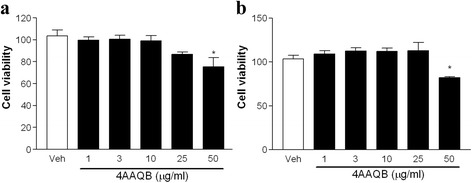
Effect of 4AAQB on cell viability of murine macrophages stimulated with lipopolysaccharides (LPS). RAW264.7 macrophages (**a**) or peritoneal macrophages (**b**) were incubated with various concentrations of 4AAQB in the presence of LPS (100 ng/ml) for 24 h. Cell viability was determined by MTT assay. Data are expressed as the mean ± S.E.M of three independent experiments and *p < 0.05 as compared with DMSO vehicle group

Firstly, we evaluated the effects of 4AAQB on cell viability of RAW264.7 murine macrophages and peritoneal macrophages using MTT cell viability assay. The cytotoxic effect was tested to find the appropriate concentration ranges of 4AAQB for the ongoing experiments. Figure 1 showed that 4AAQB has a cytotoxicity for LPS-stimulated RAW 264.7 cells and peritoneal macrophages at 50 μg/ml. We use the non-toxic concentrations (1 to 25 μg/ml) of 4AAQB to perform the following experiments.

### 4AAQB inhibits TNF-α and IL-6 production in LPS-stimulated phagocytes

**Fig. 2:**
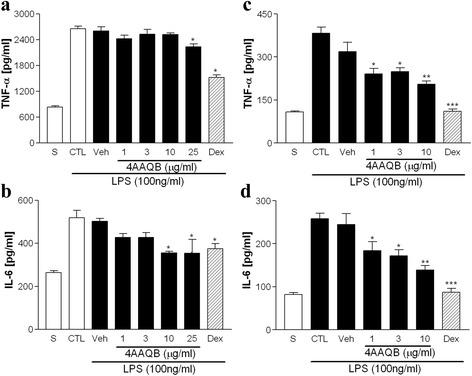
Effect of 4AAQB on TNFα and IL-6 production in LPS-stimulated macrophages. RAW264.7 macrophages (**a**, **b**) or peritoneal macrophages (**c**, **d**) were pretreated with various concentrations of 4AAQB of dexamethasone (Dex, 10 μM) for 30 min, and then activated with LPS (100 ng/ml) for 24 h. The TNFα and IL-6 concentrations were measured by ELISA kits. Results are showed as mean ± S.E.M of three independent experiments. *p < 0.05, **p < 0.01 and ***p < 0.001 as compared with LPS-treated groups

We further determine pro-inflammatory cytokines production affect by 4AAQB in LPS-stimulated RAW264.7 macrophages and peritoneal macrophages. Cells treated with LPS obviously increased the pro-inflammatory cytokines production, including TNF-α and IL-6. 4AAQB (1-25 μg/ml) inhibited the protein levels of TNF-α and IL-6 production in a concentration-dependent manner both in RAW264.7 macrophages (Fig. 2a-b) and peritoneal macrophages (Fig. 2c-d). There is no significant difference between control and vehicle groups. As a positive control, dexamethasone (10 μM) also have the ability to inhibit LPS-induced cytokine release.

### 4AAQB inhibits LPS-stimulated peritoneal macrophages migration

**Fig. 3:**
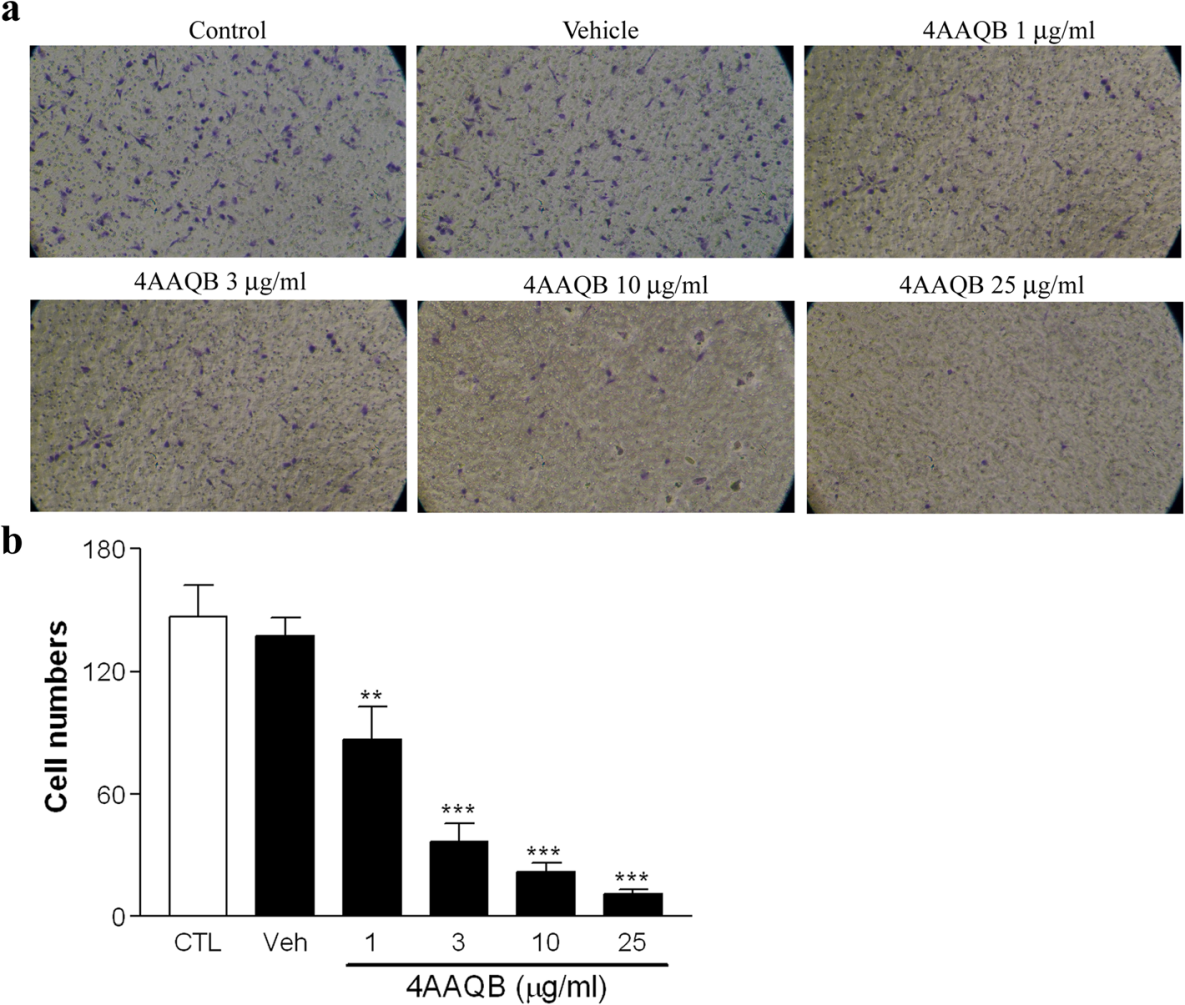
Effect of 4AAQB on LPS-induced cell migration of murine macrophages. Peritoneal macrophages were pretreated with various concentrations of 4AAQB for 30 min and then activated with LPS. The results of cell migrated through gelatin-coated transwells for 20 h were shown (**a**), and the migrated cells were counted (**b**). Data are expressed as the mean ± S.E.M of three independent experiments. **p < 0.01 and ***p < 0.001 as compared with LPS-treated control groups (CTL)

During inflammation, monocytes/macrophages enable to infiltrate from blood vessels to extravascular sites. Therefore, transwells (gelatin-coated) were used to evaluate the effect of 4AAQB on LPS-stimulated migration of peritoneal macrophages. 4AAQB displayed concentration-dependent inhibitory effect on peritoneal macrophages migration of (Fig. 3). There is no significant difference between control and vehicle groups.

### 4AAQB inhibits the inducible nitric oxide synthase (iNOS) expression and NO production in LPS-stimulated macrophage

**Fig. 4:**
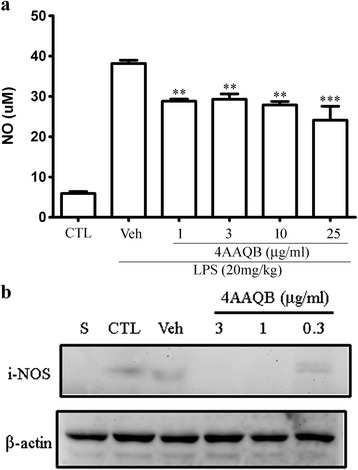
The effects of 4AAQB on LPS-induced nitric oxide production and iNOS expression in RAW264.7 macrophages. **a** The cells were treated with LPS (100 ng/ml) only or with various concentrations of 4AAQB and the nitric oxide production was measured after 24 h. Control (CTL) values were obtained in the absence of LPS. Data were obtained from three independent experiments and expressed as means ± S.E.M. *p < 0.05 compared with the LPS-activated only group. **b** RAW264.7 cells were treated with various concentration of 4AAQB and stimulated with LPS (100 ng/ml). Cells were harvested and iNOS was detected. Each experiment has been performed three times

Upon LPS stimulation, the expression of iNOS is increased, which in turn produce more amounts of NO [18]. The nitrite production and iNOS expression increasing were observed in LPS (100 ng/ml) stimulated RAW macrophages. 4AAQB treatment was able to reduce NO production (Fig. 4a) and iNOS expression induced by LPS (Fig. 4b), indicating that 4AAQB inhibits NO production and iNOS expression at the transcriptional level.

### 4AAQB inhibits the MAPK signaling pathway phosphorylation in LPS-stimulated macrophages

**Fig. 5:**
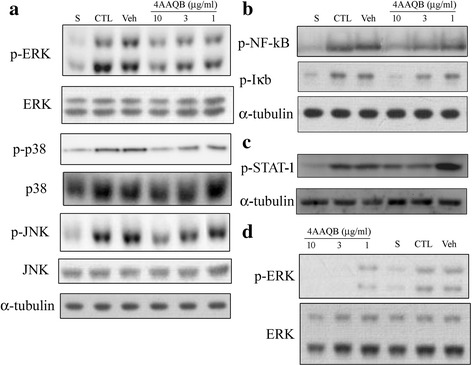
Effects of 4AAQB on the LPS-induced activation of MAP kinases, IkBα, NFκB p65 and STAT1 in RAW 264.7 macrophages and peritoneal macrophages. RAW264.7 cells were treated with various concentration of 4AAQB and stimulated with LPS (100 ng/ml) for 30 min. Cells were harvested and total cell extracts were prepared. **a** Phosphorylated-ERK, phosphorylated-JNK, phosphorylated-p38, or **b** Phosphorylated-IκBα and NFκB p65 subunit and **c** Phosphorylated-STAT1 were detected by Western blot analysis. Total ERK, JNK, p38 and α-tubulin were used as internal standard. **d** Peritoneal macrophages were treated with various concentration of 4AAQB and stimulated with LPS (100 ng/ml) for 30 min. Phosphorylated-ERK and total ERK were detected by Western blot analysis

MAPK molecules are important to regulate pro-inflammatory cytokines and NO production from activated macrophages and involved in inflammation and sepsis [19]. We further investigate the involvement of these MAPK molecules phosphorylation in 4AAQB anti-inflammatory mechanisms. The phosphorylation of extracellular signal-related kinase 1/2 (ERK1/2), p38 MAP kinase (p38) and c-Jun NH2-terminal kinase (JNK) was induced by LPS stimulation rapidly within 30 min in RAW264.7 cells and the total ERK1/2, p38, and JNK protein were not changed. There is no significant difference between control and vehicle groups. 4AAQB suppressed the phosphorylation of these MAPK molecules in LPS-stimulated macrophages in a concentration dependent manner (Fig. 5a).

### Effects of 4AAQB on NFκB and signal transducer activator of transcription 1 (STAT-1) activation in LPS-stimulated macrophages

During the inflammatory response to LPS, NFκB plays an important role in regulating the pro-inflammatory cytokines, chemokines, adhesion molecules and inducible enzymes expression such as iNOS [20]. Phosphorylation and degradation of IκBα lead to NFκB dissociate from the cytoplasmic NFκB/IκBα complex and subsequently translocate to the cell nucleus in the inflammatory process. We further determine whether 4AAQB affects the signaling pathways involved in NFκB activation. As shown in Fig. 5b, we observed that LPS-induced of IκBα and NFκB p65 phosphorylation was suppressed by 4AAQB in a concentration-dependent manner. There is no significant difference between control and vehicle groups, but the phosphorylation of STAT1 in LPS-stimulated RAW264.7 cells is inhibited by 4AAQB (Fig. 5c). Taken together, our result indicated that 4AAQB suppresses LPS-induced inflammatory response via NF-kB and STAT-1 inhibition.

### Effects of 4AAQB on ERK phosphorylation in LPS-stimulated peritoneal macrophage

To ensure the 4AAQB have similar inhibitory effect in murine peritoneal macrophage, the ERK1/2 phosphorylation in murine peritoneal macrophage was also studied. The phosphorylation of ERK1/2 was induced by LPS stimulation rapidly within 30 min in murine peritoneal macrophage and the total ERK1/2 was not changed (Fig. 5d). There is no significant difference between control and vehicle groups.

### 4AAQB inhibits pro-inflammatory cytokines and NO production in LPS-induced endotoxemia in vivo

**Fig. 6:**
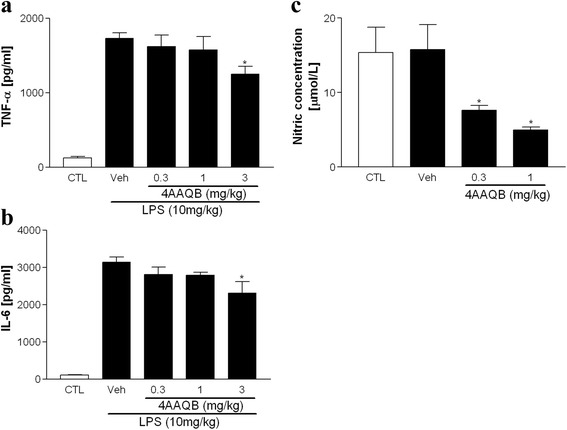
The effects of 4AAQB on LPS-induced cytokines and NO release. ICR mice (25–30 g) were treated with 4AAQB and induction of endotoxemia with LPS (20 mg/kg) after 30 min. After 24 h, mice were euthanized collect blood by cardiac puncture. The serum was obtained and the cytokines TNFα/IL-6 (**a** and **b**) and NO (**c**) were measured. Values are presented as mean ± S.E.M. (*n* = 10). *p < 0.05 as compared with the LPS-activated group

The effect of 4AAQB on acute inflammation in vivo was tested by LPS-induced endotoxemic model. Mice were injected (intraperitoneally, i.p.) with DMSO (vehicle) or 4AAQB (0.3 and 3 mg/kg) and after 30 min i.p. injected with LPS (20 mg/kg). The cytokine and NO release were measured by collected mice serum after 24 h. The LPS caused elevated plasma TNF-α and IL-6 levels were observed and 4AAQB administration concentration-dependently decreased these cytokine levels (Fig. 6a and b). The nitrite production was also reduced by 4AAQB treatment (Fig. 6c). These results indicated that 4AAQB have the ability to reduce cytokine and NO release in LPS-induced septic mice.

### 4AAQB increases survival rate of mice and inhibits pro-inflammatory cytokines production in CLP surgery

**Fig. 7:**
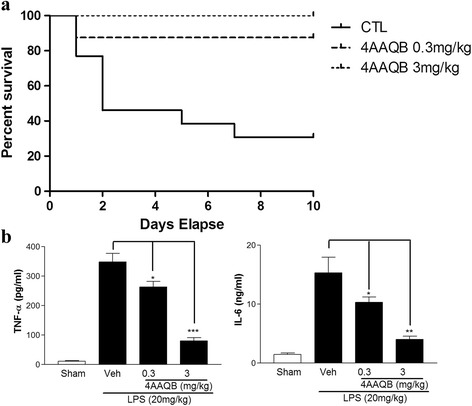
The protective effects of 4AAQB on survival rate, cytokine production and tissue inflammation induced by CLP operation in vivo. Mice were administered with 4AAQB (0.3 and 3 mg/kg, i.p., *n* = 8) in DMSO or DMSO (CTL, *n* = 13) for 30 min, and then conducted CLP operation. **a** Survival was monitored for up to 10 days. Values are presented as mean + S.E.M. *p < 0.05 as compared with the LPS-activated group. **b** Mice were anesthetized and blood was collected for serum isolation 24 h after CLP operation, and concentration of cytokines was measured by ELISA. Values are presented mean + S.E.M. and *p < 0.05 compared with LPS-treated control group

We also used another inflammation model, cecal ligation puncture (CLP)-induced septic model to test the 4AAQB anti-inflammatory effect. Mice were i.p. injected with DMSO (vehicle) or 4AAQB (0.3 and 3 mg/kg) 30 min before the CLP surgery. The survival rate was checked at indicated time point, 4AAQB administrated group notably has higher survival rate at different time points. As shown in Fig. 7a, the survival rate significantly increases in 4AAQB (0.3 mg/kg)-treated group (from 69.2% to 12.5%) and 4AAQB (3 mg/kg)-treated group (from 69.2% to 0%). The TNF-α and IL-6 release were measured by collected mice serum after surgery 24 h (Fig. 7b).

### Protective effects of 4AAQB on tissue injury in CLP-induced sepsis model

**Fig. 8:**
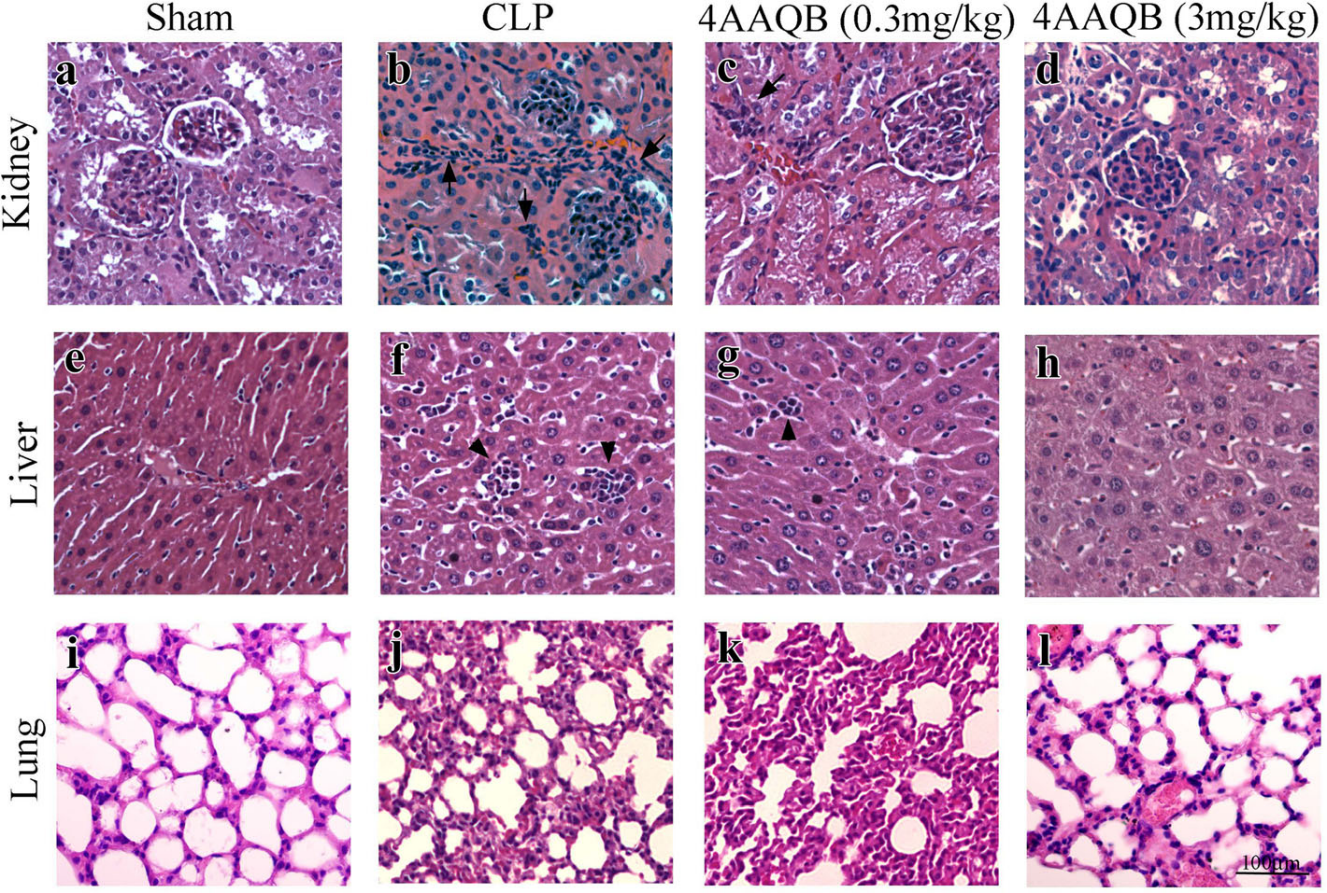
Protective effects of 4AAQB on tissue injury in a CLP-induced sepsis model. Morphological changes in the mouse kidney (**a**-**d**), liver (**e**-**h**) lung (**i**-**l**), and sections [hematoxylin and eosin (**h** & **e**) stain, all panels are × 400 with the same scale bar in panel L]. **a**, **e**, **k** control mice (Sham), **b**, **f**, **j** CLP-induced sepsis mice, **c**, **g**, **k** CLP-induced sepsis mice treated with low dose 4AAQB (0.3 mg/kg) and **d**, **h**, **l** CLP-induced sepsis mice treated with high dose 4AAQB (3 mg/kg)

The kidney in CLP-induction mice was damaged with glomerular hypercellularity as compared with sham group (Fig. 8a and b). The liver sections in CLP-induction mice also exhibited significant peri-vascular leukocyte infiltration as compared with sham group (Fig. 8e and f). The lung sections in CLP-induced septic mice was also present much higher leukocytes infiltration in the alveoli and alveolar wall thickens (Fig. 8i and j). The protective effects of 4AAQB for these organ injuries in CLP-induced septic mice were shown by in Fig. 8c, g, and k (low dose, 0.3 mg/kg) and Fig. 8d, h and l (high dose, 3 mg/kg).

## Discussion

Inflammation is a complex pathophysiological phenomenon associated with numerous human diseases. *Antrodia cinnamomea*, a unique traditional Chinese medicine deriving from Taiwan, has several reported evidences of its biological activities, especially anti-inflammatory and anti-cancer activities. This study was elucidated the molecular mechanism of 4AAQB, the component in mycelium extract of A. *cinnamomea*, on inflammation in LPS-stimulated macrophages model and LPS/CLP-induced septic models.

LPS is a well-known endotoxin to induce the activation of monocytes/macrophages, which then release inflammatory cytokines and causes septic shock [21]. Most chronic inflammatory diseases are associated with inflammatory cytokines overproduction. A previous study demonstrated that the anti-inflammatory activity of methanol extract from A. *cinnamomea* mycelia (MEMAC) via inhibiting iNOS and COX-2 expression and decreasing TNF-α and IL-6 production. Camphorataanhydride A and camphorataimide B were isolated from MEMAC and belonging to the succinic acid derivative [11]. Another study has reported that the water extract of A. *cinnamomea* mycelium inhibited the production of TNF-α, IL-1β, NO and PGE2, and iNOS and COX-2 expression in macrophages [22]. However, the active component is not clarified. In the present study, we demonstrated that 4AAQB, a component from A. *cinnamomea* mycelium, inhibits LPS-induced TNF-α and IL-6 production (Fig. 2). Furthermore, 4AAQB decreased NO production via suppressing iNOS expression (Fig. 4a). This result is consistent with the previous study [15], which has shown that antroquinonol and 4-acetylantroquinonol B (same as 4AAQB in our study) inhibited NO production and iNOS expression induced by LPS. 4AAQB is the third abundant compound in the ethanolic extract of mycelium, and possess better anti-inflammatory activity than antroquinonol, indicating that 4AAQB has the potential for use as an anti-inflammatory agent. We further clarify the molecular mechanism of 4AAQB and its anti-inflammatory effect in in vivo models.

Cell motility is one of the characteristics of activated monocytes/macrophages to infiltrate from blood vessels and migrate to the sites of inflammation to clear pathogens and defend inflammation process. Therefore, the ability of leukocytes migration is a good inflammation index. 4AAQB was shown its inhibitory effect in LPS-induced peritoneal macrophages migration (Fig. 3).

Upon LPS ligated to toll-like receptor 4 (TLR4) triggers the association with myeloid differentiation primary-response protein 88 (MyD88) and activates several intracellular signaling pathways, including MAPK pathways and IκB kinase (IKK)-NFκB pathway [23]. Treatment of 4AAQB significantly inhibited LPS-induced MAPK phosphorylation, including ERK1/2, p38 and JNK (Fig. 5a), which are the key elements for cell migration, proinflammatory cytokines production and inducible enzyme expression (e.g. iNOS). NFκB is another major pathway and activated during the inflammatory response to LPS. In resting cells, NFκB proteins are predominantly in cytoplasmic and associated with members of the inhibitory IκB to inhibit its DNA-binding activity. When cells activate, IκB undergoes phosphorylation and further ubiquitination and degradation, leading to NFκB dissociate from complex and induces an inflammatory response [20]. We found that 4AAQB inhibits LPS-induced phosphorylation of IκB and NFκB subunit p65 in RAW264.7 macrophages (Fig. 5b). STAT1 is another transcription factor involved in LPS induced inflammatory process [24]. Upon LPS stimulation, STAT1 activates in macrophages and plays a role in inflammatory process [25] and the previous study has demonstrated that STAT1 signaling is essential for iNOS overexpression in LPS-stimulated macrophages [26]. 4AAQB suppresses the phosphorylation of STAT1 stimulated by LPS (Fig. 5c) as well as iNOS expression (Fig. 4b). Taken together, our data indicated that 4AAQB exerts anti-inflammatory effects by suppressing macrophage activation via MAPK, NFκB, and STAT1 pathway.

The LPS and CLP septic model were used to investigate the effects of 4AAQB on acute inflammation in vivo. In LPS-induced endotoxemic model, we found that serum proinflammatory cytokine, including TNF-α and IL-6 were reduced in 4AAQB-treated septic mice (Fig. 6). CLP is a more clinically relevant sepsis model than injection of endotoxin (LPS). Cecum puncture causes its bacteria inside releasing and result in polymicrobial peritonitis. Bacteria translocates into the blood and lead to septic shock, multi-organ dysfunction and ultimately death [27]. Thus, CLP is considered a good method for the investigation of the pathogenesis of sepsis. We further examined the effect of 4AAQB on CLP-induced septic mice. In CLP septic model, we observed that TNF-α and IL-6 are markedly reduced in 4AAQB-treated mice, and the survival rate is also improved by 4AAQB treatment. Furthermore, 4AAQB has shown protective effect from organ leukocyte infiltration (lung, liver and kidney) due to sepsis (Fig. 7). To our best knowledge, this study is firstly demonstrated that 4AAQB exhibits anti-inflammatory efficacy in in vivo models.

## Conclusion

In conclusion, 4AAQB, an antroquinonol derivative, possesses anti-inflammatory effects in both pro-inflammatory cytokine and NO release reduction in LPS-stimulated RAW 264.7 cells via the mechanisms of inhibiting NOS2 protein expression and modulating MAPK, STAT-1 and NFκB pathways. 4AAQB also exhibit anti-inflammatory effect in LPS and CLP-induced sepsis mice model, suggesting that 4AAQB has effective anti-inflammatory properties and may be a therapeutic candidate which used in inflammatory disorders treatment.

## Additional file

Additional file 1: Structure elucidation by NMR - The assignment of the ^1^H NMR and ^13^C NMR spectrum supports the proposed structure for 4AAQB. The ^1^H NMR, ^13^C NMR spectrum were shown in **Figure S1** and **Figure S2**. (DOCX 1109 kb)

## Abbreviations

4AAQB: 4-acetylantroquinonol B
A. cinnamomea: Antrodia cinnamomea
CLP: Cecal Ligation Puncture
LPS: Lipopolysaccharide
