# Supplementary material for: 4-Acetylantroquinonol B inhibits lipopolysaccharide-induced cytokine release and alleviates sepsis through of MAPK and NFκB suppression
Source: BMC Complement Altern Med. 2018 Mar 23;18:108. doi: 10.1186/s12906-018-2172-2 (PMC5865343; doi:10.1186/s12906-018-2172-2)

Additional file 1 Structure elucidation by NMR

The results from 1H NMR, 13C NMR experiments confirm the structure of 4AAQB.

The assignment of the 1H NMR and 13C NMR spectrum supports the proposed structure for 4AAQB. The 1H NMR, 13C NMR spectrum were shown in Figure S1and Figure S2

Figure S1 1H NMR spectrum of 4AAQB


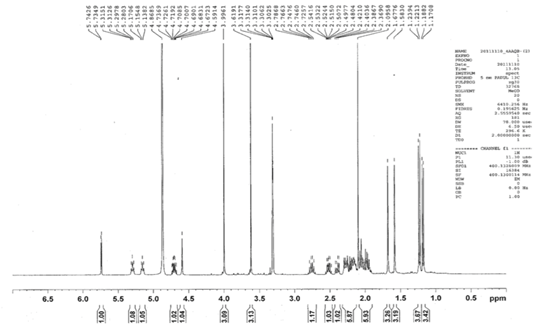


Figure S2 13C NMR spectrum of 4AAQB


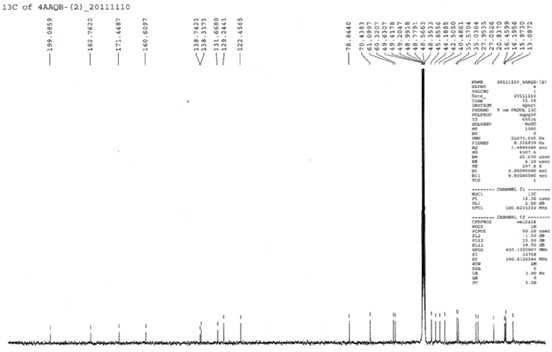

Supplement: Supplementary file 1 — Structure elucidation by NMR - The assignment of the 1H NMR and 13C NMR spectrum supports the proposed structure for 4AAQB. The 1H NMR, 13C NMR spectrum were shown in Figure S1 and Figure S2. (DOCX 1109 kb) [file 12906_2018_2172_MOESM1_ESM.docx]
